# Supplementary figures and images for: Corollary discharge enables proprioception from lateral line sensory feedback
Source: PLoS Biol. 2021 Oct 11;19(10):e3001420. doi: 10.1371/journal.pbio.3001420 (PMC8530527; doi:10.1371/journal.pbio.3001420)

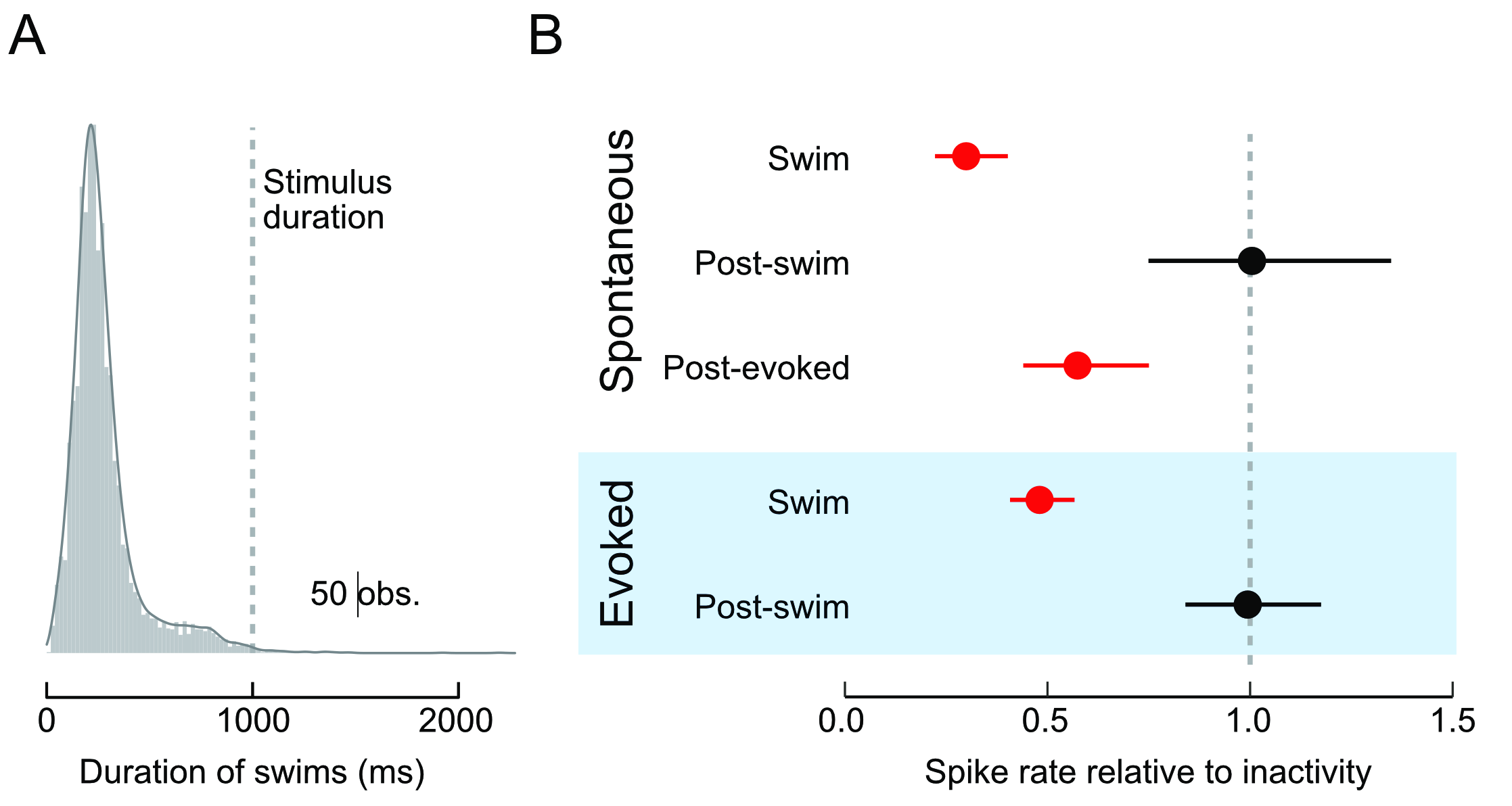

Supplement: S1 Fig — (A) Histogram of swim bout durations. (B) Average spike rate in swim (CD ON), post-swim, and post-stimulus intervals, relative to inactivity (CD OFF). This estimate of the relative evoked spike rate while swimming was obtained by averaging over all swim bouts and individuals, which resulted in a higher estimate than when accounting for differences due to cell class (Figs 4 and 6). The data and code underlying this figure may be found at DOI: 10.6084/m9.figshare.13034012. CD, corollary discharge. (TIFF) [file pbio.3001420.s001.tiff]

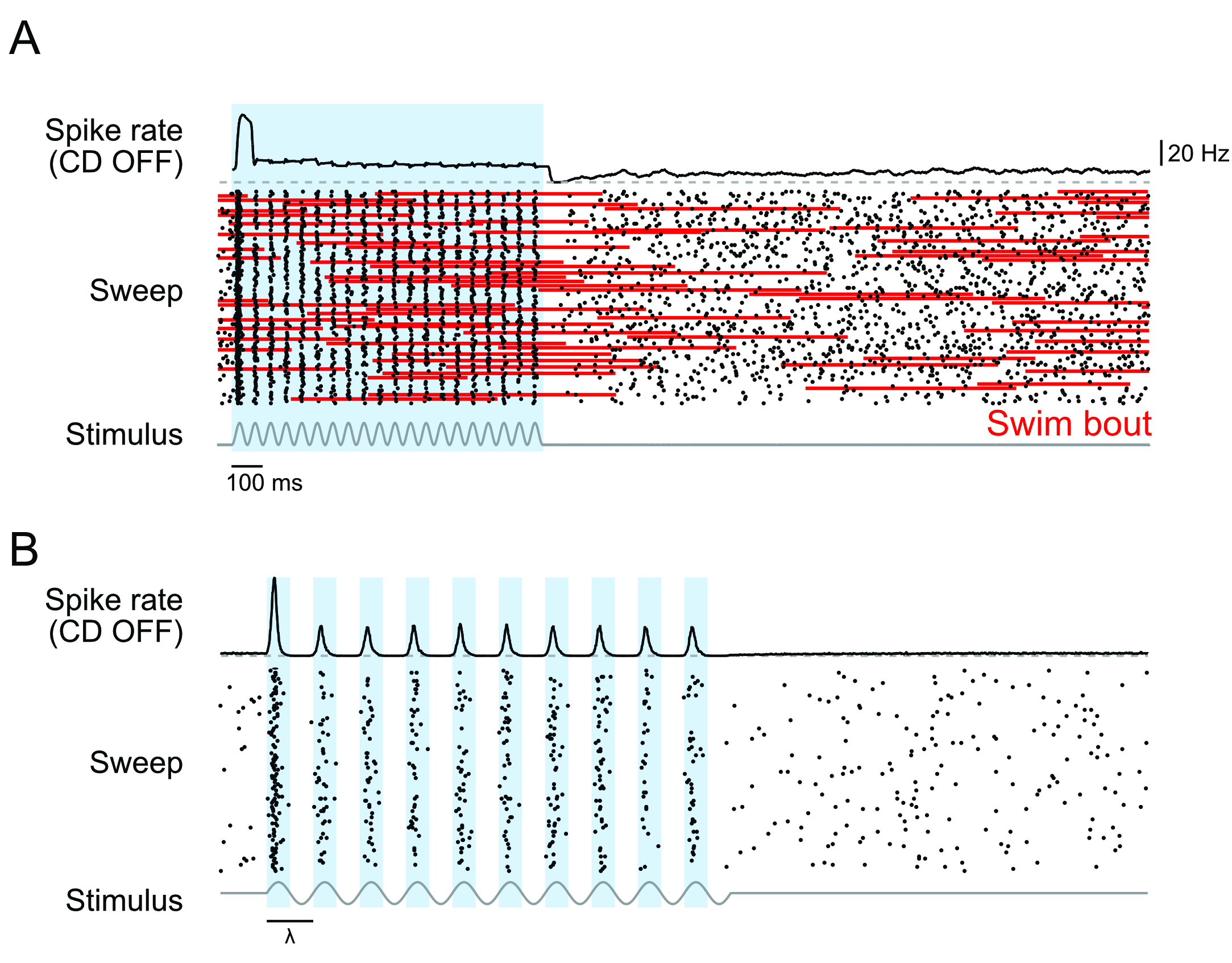

Supplement: S2 Fig — (A) PSTH at 20 Hz corresponding to individual and traces in Fig 3. Red bars denote a spontaneous swim bout. Spike rate calculated in 50-ms intervals for spikes labeled as CD OFF. Stimulus trace shown below. (B) PSTH of simulated responses. Inhibition was only applied over the entire stimulus interval. Note that responses were normalized to the wavelength period. The data and code underlying this figure may be found at DOI: 10.6084/m9.figshare.13034012. CD, corollary discharge; PSTH, peristimulus time histogram. (TIFF) [file pbio.3001420.s002.tiff]

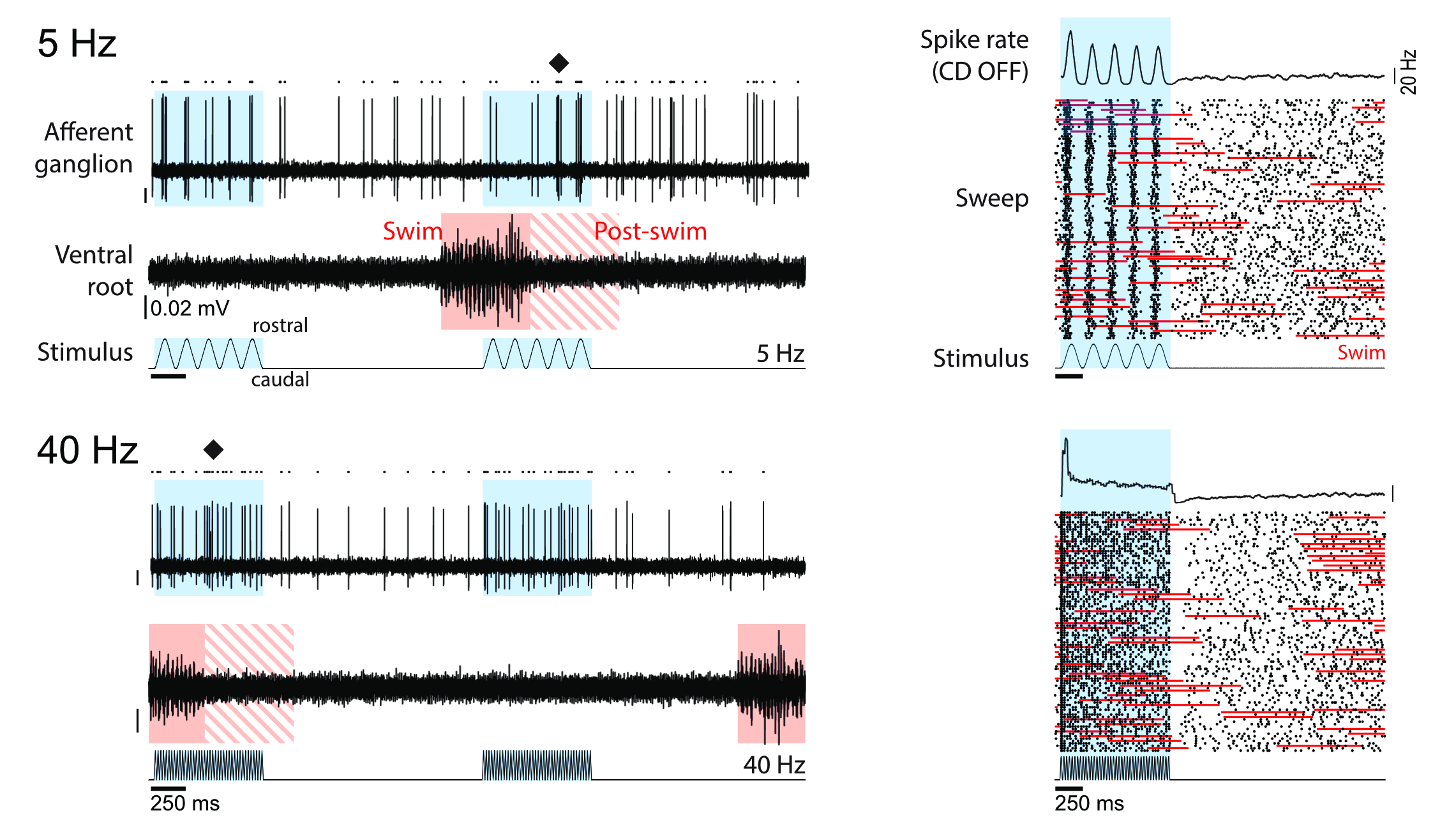

Supplement: S3 Fig — Black diamonds over afferent ganglion traces demark highly evoked responses in the immediate post-swim period (red hatched intervals, equal in duration to the swim bout). In many cases, evoked activity after a swim was equal to the evoked activity at the start of the stimulus presentation. Spike rates were calculated in 50-ms intervals. Same individual as Fig 3. The data and code underlying this figure may be found at DOI: 10.6084/m9.figshare.13034012. PSTH, peristimulus time histogram. (TIFF) [file pbio.3001420.s003.tiff]
