# Supplementary material for: Corollary discharge enables proprioception from lateral line sensory feedback
Source: PLoS Biol. 2021 Oct 11;19(10):e3001420. doi: 10.1371/journal.pbio.3001420 (PMC8530527; doi:10.1371/journal.pbio.3001420)
Supplement: Attachment — Submitted filename: reviewer comments_round2.docx [file pbio.3001420.s005.docx]

REVIEWERS' COMMENTS:


Reviewer #2:

The authors have significantly improved the presentation and clarity of their earlier manuscript in this revision. They have carried out a major rewrite and reorganization to address reviewers' concerns. I think that they have addressed all points effectively, and in particular Figures 3, 4, and 6 are tremendously easier to interpret now. The results continue to be of significant importance to the field.

I have one minor suggestion at the authors' discretion, which would be to remove the paragraph at lines 305-322 (or place it elsewhere). I found it disrupted the flow of description of Figure 3, and I couldn't understand why it was in the middle of the Results section, which is typically a more straightforward figure-by-figure presentation. However this is an optional suggestion.

We are pleased that our revisions met the reviewer’s expectations and they recognize our contribution to the field.

The paragraph referred to by the reviewer was intended to provide a road map within the body of the results. This addressed a previous reviewer concern that we had not clearly distinguished the experimental from computational results.

Upon reflection and together with the comments of Reviewer 4, we believe this paragraph created more confusion by calling the figures out of order. The paragraph has been deleted and the text moved to relevant sections. With further editing to the Results section motivated by Reviewer 4’s comments, we believe the logical progression of the manuscript will be clear to the reader.

Reviewer #3:

The authors have addressed the concerns raised by the reviewers. The manuscript is a lot easier to read and understand and the figures are simpler and easier to interpret, also the additional Figure 1 helps a lot.

We thank the reviewer for their time and are pleased they now find the manuscript more accessible.

Reviewer #4:

The authors have undertaken a SUBSTANTIAL revision here which has greatly improved the clarity and readability of the manuscript and should be applauded. I imagine this was a lot of work and as a reviewer I greatly appreciate that my review was taken seriously and implemented on such a scale. I have a few minor questions/comments/suggestions remaining.

1.      In general, I feel like I understood a lot from reading the response to Reviewer #3 particularly regarding the relationship between your work and the previous recent Lagnado papers. That was valuable to me and I would encourage you to spell that out in the manuscript explicitly. That goes for a lot of what you wrote in the response to reviewers, I feel like many of those points should be in the manuscript.

We examined our previous responses to weigh how best to position our work with respect to the work of Pichler & Lagnado. In the Introduction, we highlight that the specific goal of this work is to develop our understanding of the *interactions* between heterogeneity, adaptation, and corollary discharge, which was not done previously, and which are not straightforward to predict from the previous work. We highlight a significant difference from the previous work, which is that our current approach allows us to propose the previously unexamined role of corollary discharge on temporal coding.

*Second, we build on previous examinations of the independent effects of heterogeneity, adaptation, and corollary discharge in the lateral line (10, 49)* *by examining the interactions between these factors. These interactions are critical for understanding how sensory processing is modulated during the behavioral shifts between rest and motor activity. The proposed proprioceptive function of the lateral line is enabled by the central role of corollary discharge in regulating the magnitude and temporal characteristics of feedback from populations of heterogeneous sensors. The capacity of corollary discharge to regulate spike temporal codes as well as rate may have broad significance throughout the nervous system.*

In addition, we have ensured that the following key arguments from our previous responses are contained in the text: (1) how the choice of stimulus frequency impacts apparent responses and consequently our focus on stimulation within the natural band of fish (in Results section); (2) the potential limitations of using evoked spike rate inhibition as a proxy for corollary discharge strength (in Discussion); and (3) how our results might be related to the antero/retrograde polarization discovered by Pichler and Lagnado (in Discussion). Our discussion of the third point highlights limitations of the current study and should motivate and inform future experimental designs.

2.      Thank you for further clarifying and explaining the source of the CD and your assumptions around this. Although you state it has been extensively verified, it's still not clear to me why this signal could not be measured or manipulated directly, rather than inferred. Can you expand on this a bit? I'm not saying you should do it if it's technically challenging enough to be a whole other paper, but address it for the reader because it's not clear to me why that wouldn't be the perfect thing to do. Why not say optogenetically mess with the efferent activity? Is this possible? Wouldn't this add to your findings if these manipulations agree with your model? Just discuss please. Also I disagree that you can't do a laser ablation and then have a fish swim in a behavior or perform in a physiology experiment afterward because I've done that myself. It's annoying though, to be sure. I'm not saying you need to do it, but it's very possible and ablation + behavior experiments have been published since Liu and Fetcho 1999.

We are in complete agreement that behavioural assays coupled with optogenetics will be essential for validating the model we have put forth. This was not possible in the current work due to numerous technical challenges that would make inference challenging.

Our retrograde tracing inevitably resulted in highly variable numbers of labelled cells in the hindbrain, even when performing bilateral injections on the same animal (between 2-6 neurons). This would result in unequal numbers of ablated cells in the left and right hemispheres, and therefore uncontrolled behavioral defects that would be unlikely to shed any light on our model. Added to this, the spatial targets of efferent neurons in the hindbrain are not currently known; by chance, we could end up labeling and ablating caudal targets on one side and rostral targets on the other. Altogether, the invasive nature of retrograde tracing and ablation together with its inherent uncertainty would be difficult to interpret.

As pointed out, a better tool may be optogenetic dissection. Unfortunately, no distinct molecular marker of the efferent cells has yet been discovered. The cells themselves sit in dense cholinergic cell clusters in the hind brain, which we have previously visualized with the Islet promotor [e.g. Higashijima et al 2000. J. Neuro. 20(1):206]. Without a distinct promoter, optogenetic modulation would cause widescale disruption of the entire cholinergic network, and inevitably affect swimming.

We further considered the possibility of reversibly blocking hair cell acetylcholine receptors. However, the available nAChR blockers reversibly bind hair cell receptors while irreversibly binding the muscle receptors, which prevents swimming but not the inhibition.

As we believe the reviewer kindly agrees, a complete review of these challenges is beyond our scope. To maintain focus and brevity in the spirit of previous reviews, we simply point out that optogenetics will be one tool within the broader need for studying the context of free behaviour:

*A critical next step for understanding the role of reafferent feedback in motor control will be to unravel the nature of the controller in the central nervous system (Figure 1 B i). Studies of freely behaving fish will be essential, likely including optogenetic dissection of the CD’s role in swimming feedback control and natural swim motions. Similarly, the actual magnitude and temporal dynamics of cupular deflection during swims is unknown, even though the coherence and encoding capacity of lateral line feedback critically depends on the input signal parameters (8, 23).*

3.      The figures are much much MUCH clearer but they are still a bit in a roundabout order (Figure 3A, Figure 4, Figure 5, Figure 6, Figure 3B, Figure 4, etc etc) so I suggest either discussing them in the order presented or presenting them in the order you mean to discuss them.
Upon consideration, this appears to principally be an issue of the paragraph identified by Reviewer 2. We now see that that paragraph caused confusion with respect to the order of presentation.

We have deleted this paragraph and moved each of the elements to their logical place within the text. In our reading, this has entirely resolved the order of figure call-outs, except when previous figures are used to provide further context to the current one, especially to justify more detailed analyses or the adequacy of the computational models.

4.      Re Figure 5, it wasn't clear to me why you only looked at post swim and not also pre swim.
A fundamental goal of corollary discharge research is to examine how sensory processing is affected during and after a locomotor event. In this system, we have not seen any evidence of a pre-swim preparation that can be identified from the spike code, nor was such an effect seen in our previous analysis of spontaneous spike rates. Given our knowledge of the system’s circuitry and dynamics, it is not clear how a pre-swim signal could occur.

Our previous work did reveal long inhibition time constants that we associated with acetylcholine washout during the transition from swimming to rest. This longer washout might reduce sensitivity to evoked stimuli in the post-swim period, and therefore motivated our examination of the swim and post-swim periods.

We have added additional context for the reader at two spots in the Results:

[added text in blue] *Importantly though, the function of CD to inhibit vesicle release will protect the vesicle pool from depletion so that it is primed to robustly respond to flow perturbations after the swim. On the other hand, as discussed below, excessive inhibition by the CD can also introduce post-swim artefacts. We therefore paid special attention to the effect of CD, stimulation, and sensor heterogeneity on spike properties through the swim bout and into the post-swim period.*

*The rapid restoration of evoked spiking suggested that CD protects sensors from adaptation and from being desensitized to stimuli that occur in the post-swim period. On the other hand, we previously found a persistent suppression of spontaneous spiking after the swim that could reduce sensitivity (48).*

In the methods section, we added the following clarification,

*These two intervals are of principal interest in studying the consequences of movement on sensing. Modulation of the pre-swim period was not revealed in our previous analysis of spontaneous spike rates in the pre-swim period (48).*

5.      My main issue with this paper is still the readability, even though it must be said it is DRASTICALLY improved from the previous version. I often found myself in the middle of a results paragraph saying "why are we doing this?" or at the end of one saying "what is the take home here?" I would recommend more concise guiding sentences for the reader. There is an absolute ton of complicated analysis here and while it's now possible for me to understand it (yay!) it's not always clear why this is being undertaken, what is being learned and gained, from each plot or figure. Perhaps that's just that this isn't my exact area of work (as you can tell) but I'd imagine you'd like this paper to appeal to folks like me.

We have endeavored to read each paragraph of the results to ensure the first and last sentences encapsulate a goal and take-home message. As far as statistical analyses go, we believe that through the review process we have effectively narrowed and simplified the analyses only to what is necessary for drawing our conclusions.

There are also still labels and descriptions missing on the figures and legends so please review carefully. If you're skipping axis labels for an aesthetic reason I would suggest against this as it was a bit confusing especially in cases like Figure 4. If the reader is less annoyed they will be more likely to read the whole thing.

We reexamined all figures and added all omitted labels and descriptions to Figures 4-7, as suggested. We additionally corrected the y axis units of Figure 6 ii (+0.5 had erroneously been added to the positive axis values).

We have endeavored to remove unnecessary text and clarify the key points in each figure title and caption.
