# Supplementary material for: Corollary discharge enables proprioception from lateral line sensory feedback
Source: PLoS Biol. 2021 Oct 11;19(10):e3001420. doi: 10.1371/journal.pbio.3001420 (PMC8530527; doi:10.1371/journal.pbio.3001420)
Supplement: Attachment — Submitted filename: reviewer comments.docx [file pbio.3001420.s004.docx]

Reviewer #1:

[identifies himself as Leon Lagnado]

Skandalis et al.

Corollary discharge prevents signal distortion and enhances sensing during locomotion

This paper addresses a general and important issue in sensory neuroscience: how does the nervous system deal with stimulation of sensory systems resulting from the animals own motor behaviour rather than events in the external world. This issue has been investigated in a number of contexts, such as the fly visual system, but here the authors investigate mechanical sensing in the lateral line of fish, where it has long been known that motor activity is associated with a copy of the motor signal (a "corollary discharge") delivered directly to the primary receptors (hair cells) through efferent fibers in the sensory organ (neuromast) that senses water flow over the body (effectively a form of proprioception). It has long been known that this CD inhibits hair cell activity and recently it was demonstrated that this inhibition is to a large degree selective for the hair cells that are polarized to be most strongly activated during forward swimming motion. This work now links this information into a bigger picture by developing "a data-driven model integrating swimming biomechanics, hair cell physiology, and corollary discharge to understand how sensory modulation is calibrated during locomotion in larval zebrafish". The authors use this model to test the hypothesis that a major effect of the CD is improve the temporal accuracy with which the amplitude and speed of undulatory body motions are signalled by the lateral line. This hypothesis is based on the idea that the CD will counteracting the phase distortions caused by the heterogenous rates of depressing adaptation in different hair cells within the neuromast and narrow the time-window over which afferent fibers are active by increasing the threshold for activation of synaptic transmission from hair cells.

This is an excellent paper which makes a decisive contribution and deserves a wide audience. It is conceptually rich and does an excellent job of integrating hair cell biophysics into the bigger picture of the lateral line in action. The central hypothesis is interesting and reasonable and the experiments designed to test it are unusually direct, demonstrating that CD does indeed improve timing accuracy in at least some afferent fibers. The discussion of the results is nuanced and thoughtful and again emphasizes the wider functional role of the lateral line system.

We thank the reviewer for their kind comments and we are pleased they appreciated the goals and intents of our contribution.

Major criticisms: None. The experiments are designed well and analysed appropriately. The interpretations of the results are reasonable. Wider discussion of results are also balanced and reasonable.

We thank the reviewer again and hope that the revisions meet the same standards.

Minor comments

Line 168: Fig. 1Ai and ii should be Fig. 2Ai and ii I think

Figures and text have been reorganized. Errors with figure labels and references were also caught by other reviewers, and so we have endeavoured to more rigorously check correct figure references in the revision.

Fig. 3C: what are the values of the scale bars? Are these averages over time? Please make legend clearer, I had trouble with this.

Thank you for catching this, the values were erroneously omitted while editing the figure.

Reviewer #2:

In this manuscript, Skandalis and colleagues present a new analysis of the role of corollary discharge in mitigating self-generated sensory stimuli (reafference). Their central idea is that corollary discharge effectively narrows the temporal window for sensory inputs so that (a) excessive responses are diminished, and (b) the responses that do occur are tightly timed with respect to the body bend, permitting functional proprioception.

This idea is very interesting and speaks to a vital topic in the field of motor control, and their data and analyses seem compelling. The concept of the role of motor feedback being to tighten sensory responses and limit habituation is quite powerful. At the same time, the presentation of the data was very difficult to follow, and the final model in particular was baffling as it seemed to combine both hair cells and afferents into a single element. If the authors are able to address these concerns, this manuscript would be of interest to a broad audience.

We thank the reviewer for their insight into how to make the manuscript more broadly readable and compelling. We have completely reorganized and rewritten the manuscript so that the results are presented in a more logical flow that will be more accessible. We have added a new first figure that should assist the reader in understanding the justifications for all our models. We have also substantially expanded on our justification for the final models, including a discussion of the limitations.

In brief, our measurement of spike activity in afferent neurons subsumes all upstream biophysical heterogeneity. However, the model itself is completely general and does not depend on the details of the cause of habituation. We have revised the model to more clearly show this generality and not to refer to specific elements of the hair cell system, such as the vesicles. We hope instead that the model will spur future research to identify the biological components of the model parameters.

Major issues

1. There are many comparisons made between various aspects of the data, and they are presented at too extracted a level (initially) for readability. Evoked vs spontaneous, early vs late in a train, CD or no CD, four different groups, and frequency of stimulation all make their first appearance in Fig 2. It's hard to know what to focus on. This reader, at any rate, would benefit if these were introduced more slowly, perhaps with an early figure exclusively covering habituation during CD off periods, and a later figure showing the effect of swim and/or frequency, or some other order. The subtraction of delta S and delta E (2Bii) seemed unnecessary, since those data are already compared in 2Bi, and the use of delta S, delta E, delta % (for habituation), and delta CD was taxing to keep in mind.

We have completely revised the flow of the presentation and analysis with this critique in mind. Our new Figure 3 focuses only on representative data traces to initiate the reader to our parameters. We have also clarified in the text the motivations and calculations of the parameters. Figure 4 presents only the average effects of habituation and corollary discharge, and introduces the relative comparisons used later. Figure 6 now presents all of the data concerning the differentiation of cell classes. We hope that by presenting the data in this sequence, the logical development of the paper will be much clearer to the reader.

We replaced all variables with delta symbology by more easily interpretable activity ratios.

2. A key claim in the results is that the effect of CD is to narrow the time window for evoked stimulus responses. In the examples presented in Fig 3A, however, there are a lot fewer responses for CD on than for CD off (red dots compared to gray). I was skeptical that the narrower distribution seen in some of these plots actually reflected a narrower response range, because fewer points will undersample the range. Can the authors present a statistical comparison of the variance during the two conditions?

The reviewer observes an excellent point that was a key factor in our statistical design. The combination of habituation and CD ON can result in a low probability of spiking in any particular interval. This is because the probability of a spike is the product of both the probability of a swim and the probability of a spike within a swim. For spike counts, this is not an issue because the absence of a spike is a true zero, but for the temporal measurements it is missing data. To make matters worse, this missing data is *correlated* with the effect that we seek to understand (i.e. the effects of corollary discharge and adaptation).

This problem is dealt with in two ways. First, although we show here the first and last intervals for clarity, we find the smooth function that fits through the entire time sequence. At 40 Hz, this means that the actual changes in responses are fit to 40 intervals. The effect of swimming is then determined as an offset to this average behaviour, i.e., an intercept difference. Thus the analysis of the sequences actually uses all available data within the recordings, not just the data available within single intervals. Moreover, this statistical design is actually necessary because in an open loop experiment in which the swim phase is not controlled, we cannot resolve specific effects of CD on a specific interval (this is discussed further in the text).

The variances of the smooth functions are shown by confidence bands in Figures 4 and 6, and the variance in the parameter estimates is shown by confidence intervals around the point estimates in Figures 4 and 6. The variances of parameters like the average effects of adaptation and CD across frequencies were estimated by simulations from the posteriors of each model.

These aspects of the statistical model design have now been clarified in the text.

3. The authors separate the recorded afferents into four groups. Group IV only has two cells, both of which were recorded at 5 Hz, which seems to make for a less compelling group identity. How important are these groups? The authors do not discuss whether any of these groups correspond to afferents with the opposite directional tuning, which are not subject to the same suppression during swim (Pichler and Lagnado 2020). The authors state that they could not determine directionality of responses in the Methods, though I was unclear why. The effects of CD on all four groups aren't ever broken out, as best as I can tell, in Fig 3, which raises the question of why divide into those groups in the first place.

Our goal was to show that differences in levels of adaptation lead to differences in afferent response properties during inactivity and with swimming (CD OFF and ON, respectively). The clustering analysis is now revised to correspond just to the three groups that are visually identifiable based on responses to CD, which eliminates group IV. This simplifies both the presentation and interpretation.

The core groups do bear a compelling similarity to those responses identified by Pichler & Lagnado (2020). To calculate preferred phase, we would have needed to stimulate hair cells at very low frequencies, which we unfortunately did not do at the time of data collection in 2019. At higher stimulus frequencies than were studied by Pichler & Lagnado, spike phases exhibit characteristic phase changes due to several factors including linear conduction delays within afferent fibres. We are not confident in our ability to compensate these effects with our data. Among other problems, the phase is not constant over time, but correlated with the level of adaptation. Altogether, because our experiment was not designed to discriminate polarization, we decided that errors could lead to misleading results and have therefore refrained from attempting to do so. Clearly, this will be an important parameter to quantify in future work, but we do not believe it affects the novelty of our findings on the interactions of CD and adaptation.

4. The model presented in Figure 5 appears to conflate hair cells and afferents. Throughout the manuscript, the authors have been referring to vesicle release from hair cells, so that was what I thought was being modeled here—but then they speak of spiking, whereas hair cells are non-spiking. Thus it seems this is a model of afferent release of vesicles, but I don't understand how that can be connected with the presented data, since the authors aren't recording from neurons downstream of afferents. Presumably I'm missing something here.

Our language was imprecise when presenting the model and the description overstepped the actual parameterization. The model was motivated primarily by the goal of demonstrating the effects of adaptation in sensory responses, which are encoded as afferent spikes. However, the model itself does not depend on specific assumptions about the biophysics of adaptation. For example, there could be post-synaptic inhibition in the synaptic cleft that would not be modelled by hair cell vesicle release, but which would not ultimately alter our computed behaviours.

We have added a new figure 1 that we hope will clarify the relationships between CD, hair cells, and afferent neurons that we consider here. We have also clarified the text and the figure (now Figure 7) pertaining to the computational model to show that the hair cells and afferent neurons, collectively modelled as a ‘lateral line sensor’, transition between sensitivity and insensitivity.

5. There were a number of confusing or incorrect elements in the figures. (a) There seemed to be a mismatch in the data presented in Fig 2Bi and 2Biii. There is a large orange circle in 2Bi, representing >2 spikes difference in early vs late spiking, but there is no equivalent height dot in Fig 2Biii (it should be up near the height of 2x the scale bar, if I understand correctly.)

We thank the reviewer for their careful inspection and spotting this error. We regret that the colours of two groups were inadvertently reversed while editing figures. This was not caught in our initial draft because the responses of these two groups were otherwise the same, and so the error did not affect our conclusions.

(b) What are the red lines in Fig. 2Aii? In supp Fig 1 these appear to represent periods of swim, based on the red "swim" label, but this graph is explicitly labeled CD OFF so that can't be correct here.

The reviewer is correct, the red lines depict swims. The CD OFF label was intended only to refer to the depicted mean spike rate, but we now see how this was confusing. This panel has been moved to supplementary, and the figure caption clarifies this.

(c) Is there any meaning to the vertical spacing of dots in Fig 3A? They seem oddly distributed, e.g. sometimes bimodal, and I couldn't tell if there was a reason for this.

The vertical spacing represents the sweep, from bottom to top, which has now been made explicit on the figure. We have also clarified in the text that the odd spacing is actually a consequence of the fact that we did not use a visual stimulus while recording, which causes larval zebrafish to periodically ‘give up’ on swimming (Mu et al. Cell 2019).

(d) What are the two rows of data in Fig 3Div? First and last spikes? It's not described in the legend.

The figure showed the measured phase quantiles for each group. This has been replaced in the new Figure 6 by a more intuitive depiction of the response intervals, and we have made certain that the different phase quantiles (0.1, 0.5, and 0.9 q) have been indicated on the figure.

(e) "Sample sizes as in Fig 1" (Fig 3, legend) presumably should be "Fig 2"?

Thank you for pointing out this error, which was introduced when Figure 1 was added in a previous version.

Minor points

1. The abstract sounds as though this manuscript is only a model, and doesn't contain new data. Please revise to alert readers to the experimental setup and results.

Thank you, we have thoroughly revised the abstract, and kept this detail in mind.

2. It was a bit difficult to understand why early spikes would be delayed and late spikes advanced by CD, line 135. Can the authors clarify if this is an iceberg effect or something else?

We had not previously heard of an iceberg effect. If we consider the portion of the iceberg visible above the water, then sinking a triangular iceberg will show a symmetrically smaller portion of the tip. This could indeed be an analogy for the situation with CD ON. However, the normal adapting response of CD OFF is perhaps more like a tipping iceberg because the sinking also causes the tip to shift and roll over. Unfortunately, we were not able to develop this metaphor in a way that would increase clarity for a reader.

We have better explained this phenomenon with the following text:

*When the cupula is deflected from rest along its preferred axis, spiking will be reliably evoked only when the deflection passes the higher threshold set by CD, i.e., the hyperpolarization offset. This results in a delay of early spikes. During the falling phase of the stimulus after the peak, spiking can be evoked until the cupular deflection again passes the CD-induced threshold. As the level of hyperpolarization increases, the delay of early spikes and advance of late spikes will shrink the response interval. Eventually, sensory responses will be restricted to only the strongest phase of the input stimulus, and thus will occur in a narrow band (response interval) centered on π/2. The change in threshold due to CD is therefore equivalent to sharpening lateral line sensors’ polarization by rejecting sub-threshold stimuli.*

3. In Fig. 2Aiii, various periods are labeled as delta S. However, I thought delta S referred to a subtraction of the spont data during swim minus the spont data outside of swim? This could be more clear.

We agree. Variables previously reported with a delta symbol have been replaced with more straightforward ratios of activities between CD ON and CD OFF.

4. Line 191, does "over time" mean from early to late in the stimulus train?

This is correct, but the specific line has been rewritten.

5. Line 219-220, "Habituation had a small effect on spike count…" doesn't appear to be what's quantified in Fig 2B, and there is no Fig 2C. Perhaps Fig 3 was intended?

We apologize for this error, Figure 3 was indeed the correct reference.

6. Line 241, should CD OFF be CD ON?

This is correct. The section has been rewritten.

7. At the end of the swim bout, fish are still gliding forward. Can the authors comment on what this means for their post-swim data? Is it irrelevant because the forward motion is insignificant for the lateral line sensors?

This is indeed the case and was dealt with in depth in our previous manuscript (Lunsford et al 2019). We have added text to acknowledge this,

*Such artefacts could be smoothed out by the continuous post-swim decline of both stimulation and inhibition after the swim (46), as opposed to the abrupt changes between CD ON and OFF modeled here (Figure 7 B).*

8. Line 269, what is "significance of smooth" both here and elsewhere? What statistic is this?

We have revised the Methods to better explain the parametric and non-parametric portions of the modelling. The smooth fit is the non-parametric part, and we state,

*The statistical significance of this smooth term indicates whether a nonlinear fit contributes to explaining patterns in the data.*

As the analyses are actually Bayesian, there is no test statistic for comparison. Significance is derived by examining the posterior intervals of the fits. This is all performed internally in the R package mgcv, and we cite the relevant literature discussing how this is done.

9. The presentation of data in Fig 3Div was very challenging. Are these all percents? Delta (%) suggests yes, but then how can anything be suppressed more than 100% (per the scale bar)? Is it possible to show the effect of CD on the same plot as the left column, so that the calculation of percent CD is less complicated to interpret?

We have completely revised this figure (now Figure 4) with an aim to better conveying the information. We report a more straightforward ratio of activities.

Reviewer #3:

Skandalis et al investigate the role of corollary discharge and how it affects sensing external and self-generated stimuli in the zebrafish lateral line. They record from afferent neurons during stimulation of the lateral line and, by clustering, find four differently responding groups of neurons. By analyzing the different properties and modeling they claim that corollary discharge is an important feature in fast moving species as it prevents habituation of lateral line hair cells and therefore preserves sensitivity after swimming and prevents phase shifts. Unfortunately, this manuscript is extremely difficult to evaluate as the key findings are often insufficiently highlighted and the analyses are difficult to follow. It is therefore hard for this reader to share the manuscripts' conclusions. Most of the manuscript would benefit considerably from paragraphs with topic sentences and conclusions. Frankly, it is hard to discern what the major advance over work by Pichler and Lagnado 2019 and 2020 are; those data and conclusions — particularly about different subtypes of lateral line cells and heterogeneity — are highly similar and the Discussion here does not deal with that aspect. Finally, the authors are encouraged to look into recent work from John Tuthill's group and other colleagues examining the intersection of proprioceptive signals and movement; treatment of that literature in the Discussion would broaden the target audience.

We regret the lack of clarity in our prior version. We have endeavoured to thoroughly revise the text to better highlight our contributions. We have added a new Figure 1 to highlight our first major contribution in developing a model of how proprioceptive feedback arises from the biomechanics of undulatory swimming.

Pichler and Lagnado (2019,2020) made great insights into both adaptation and CD of the lateral line but did not examine the interaction between them. This is a fundamentally important advance because, as we show, it is not straightforward to predict lateral line feedback from each alone. Moreover, we place this within a very wide context of proprioceptive feedback. The capacity to extract proprioceptive feedback from lateral line spikes was previously discounted. We now develop a theoretical model with computational support that shows precisely how information can be extracted, which we expect will spur new research on this fascinating phenomenon. We are confident that our results therefore both build on and go beyond the previous work.

As the reviewer suggested, further review of the literature on proprioception, including that of Tuthill and colleagues, has yielded several important insights that have helped us better position our work within the literature. However, aquatic locomotion is fundamentally different from jointed locomotion that has been an intensive model for proprioceptive feedback. Relatively little is known about proprioception in fish, which is better described as a continuum joint. This is a major class of joint of rapidly increasing interest in a variety of fields. We have therefore surveyed and cited a broader literature, including soft robotics, which should increase the manuscript’s appeal to a broader audience.

Fig 1Bi should say "discharge"

Thank you.

Fig 2 it's really not clear what "first" and "last" refer to — if the reader looks at the figures there's no chance they will understand what is going on without a deep dive into the text — maybe a visual indicator that "first/last" refer to the stimulus?

We have endeavoured to highlight these intervals at many points in the text so that it is clear to the reader. The revised neuromechanical model in Figure 2 highlights First and Last from the swim to the stimulus intervals, and this is carried forward through the figures.

Figure 2 Bi does not make a strong case for segregating groups 1&4 from one another.

We have revised the cluster analysis to more clearly develop the logical flow of the analyses. We use a different clustering approach that finally returns 3 groups.

Figure 2 B what do a,b, and c refer to? Is there some relationship to the letters in Figure 3, which are similarly not described?

The letters were used to denote significant statistical differences among groups. To maintain visual clarity, they have been removed in the revised figure versions. Statistical significance is summarized in a supplementary document.

The abstract suggests that this is the main advance of the paper:

"Activation of the corollary discharge prevents habituation, reduces response heterogeneity, and regulates response phases in a narrow interval around the time of the peak stimulus."

But there is not clear evidence for these claims in Figure 3D — even given the manuscripts' segregation of afferent cell types. This is also decidedly frequency-dependent with very different consequences at 5 and 20/40Hz, suggesting that for the nervous system to make sense of this change in habituation it would also be dependent on the speed of the tail movement. The manuscript seems to agree with these points per 244-251 so why highlight these findings in the Abstract when they aren't central to the paper?

We respectfully disagree with the reviewer that these are not central to our findings. It is true that CD does not render the responses identical among all afferent units. However, the responses *over time* become homogeneous and consistent because the nonlinearities are eliminated and on average, the responses are centered on the peak. We believe this is a major finding, given that it is increasingly clear that high-precision motor systems in fluid environments rely on temporal coding (Goulet et al., 2012; Putney et al 2021).

The differences between frequencies can be predicted from the interactions between CD and adaptation. Higher frequencies result in greater levels of adaptation, so it is unsurprising that CD has different effects between 5 Hz (little adaptation) and 20/40 Hz (large adaptation). This observation is central to our contention that researchers must carefully consider how the stimulus frequency corresponds to the expected reafference frequencies. This argument has been moved from the Discussion to early in the Results so that the reader has a better understanding of how we reached this decision.

In Figure 3 C and D i-iv: what do the letters above the circles mean?

As above, these referred to statistical differences between groups. The letters have been removed for visual clarity.

Line 83: Because of which arrangement, precisely?

This passage has been clarified.

Line 133: What, precisely, is meant by "vector strength?"

Vector strength is a common measure of the phase locking of a sensory neuron to a periodic stimulus. We have substantially revised the text to introduce the main variables we measure (spike count, spike phase, vector strength, and gain), and to better explain our motivation from the literature in studying these variables.

Line 219: What does "the smooth functions" refer to?

Smooth functions are the non-parametric statistical splines used to fit continuous data through generalized additive models. An alternative could be to calculate point-wise differences among groups, but this would inflate the degrees of freedom.

Line 276-278: Why would you need CD (a signal that modulates with the swim command) to do that, rather than simply tonic inhibition during movement?

We are not confident that we have fully appreciated the reviewer’s meaning. CD refers to a broad class of signals that induce a wide range of gain modulatory effects, including tonic inhibition. The CD appears to be modulated with the swim command insofar as it is a parallel copy (Pichler & Lagnado 2020). Nonetheless, to the best of our current understanding, the temporal dynamics of efferent inhibition are tonic because the time constants of acetylcholine washout (which persist post-swim; Lunsford et al 2019) are long compared to the interspike intervals of the motor command (Pichler & Lagnado 2020). The point we wish to convey here is that naturalistic activation of the cholinergic fibers during a swim bout results in the same charge accumulation previously found while galvanically stimulating the cholinergic fibers. We have clarified this text and demonstrated this effect computationally in a new panel in Figure 7.

Line 281: should be declines

Thank you. The text in this section has been thoroughly revised.

Line 341: Given the claims in Figure 1, and the return at the end of the vesicular model to this point, it would be really useful to be explicit in precisely how much worse the "estimate of the body wave" would be as a function of CD strength. Absent that, it's really difficult to determine how important CD might be for the proposed computation it is supposedly influencing (eg line 361 "a vital role for CD.") Such a computation would constitute a meaningful step forward from the model/framing of Pichler and Lagnado.

We thank the reviewer for this suggestion and have added a new analysis to our computation model. Our work certainly builds on the data of Pichler and Lagnado. However, those authors studied hair cells alone, not afferent neurons. Feedback from the lateral line is ultimately encoded by spikes. We therefore simulate spike feedback from a population of heterogeneous lateral line sensors to examine the role of CD in performing the peak detection computation proposed here. We show that unmodulated lateral line feedback is difficult to discriminate even from spontaneous feedback. However, strong modulation greatly increases the capacity to determine parameters of the reafference, including its frequency. We discuss how the biology of the lateral line, including afferent connectivity and input resistance, can further increase the robustness of peak detection. We are not aware of any other research that has proposed that CD enables computations within microcircuits like the lateral line. We are therefore confident in the novelty of our approach and findings.

Line 395-397: Could you explain a bit more what the function of Group 4 cells could be?

In our revised analysis, group 4 responses have been subsumed into group 2, which represents responses with a mixture of adaptation levels. This approach is more conservative and is supported by our computational simulations that show that we expect to observe a mix of adaptation rates with low levels of inhibition. We have revised our text to include a better description of the possible roles of each type of cell, in light of the findings of Pichler and Lagnado.

Line 469: it seems like there is a part of the sentence missing

This text has been revised and is no longer relevant.

Reviewer #4:

This paper is centered on a very interesting phenomenon of high importance, namely understanding how sensory feedback from the mechanosensory lateral line contributes to the control of swimming in fishes. To do this they use an excellent model system, the larval zebrafish, which is amenable to electrophysiological recording in conjunction with lateral line stimulation. The Liao lab is a leader in this field and the technical expertise in these types of experiments is very strong. This is a question of high interest to the field of motor control, understanding proprioception's role in general, and it has been particularly poorly understood in fishes. Therefore, this work is very important and applicable beyond the zebrafish to sensory-motor integration in general.

There are several difficulties in the presentation of the manuscript which require minor revisions in my view. I am strongly motivated to understand this paper and yet I had great difficulty. Despite mostly clear introductory text, I found some general critiques or some components either missing or not clearly communicated for the non-expert.

1) The title of the paper starts with Corollary discharge (CD) yet the actual neurons responsible for providing this CD are not discussed for 1.5 pages, and then barely at all. What happens to swimming or to afferent firing if you remove CD entirely by some ablation or lesion? Has this been done? If so, please discuss. If not, can you do it here to test its role in your model?

We structured our Introduction from general to specific concepts to ensure the manuscript’s relevance to a broad range of readers. Corollary discharge is a conceptually broad topic and in our opinion, more important than the identities of the cells that carry the CD in this specific case. For this reason, we first develop CD for a broad audience and subsequently discuss the specific cellular mechanisms of the CD to the lateral line.

We previously performed laser ablations of the hindbrain cholinergic neurons in our previous work (Lunsford et al 2019). We found that ablation of the CD-carrying cells likewise abolishes the suppression of spontaneous feedback.

*The CD is strictly activated during swim bouts (33, 46, 47), and laser ablation of the cholinergic cell bodies prevents inhibition (46).*

In our previous work we did not examine the effect of the laser ablation on the swim bout per se because fish were paralyzed for the operation. This procedure would introduce many confounding effects that prevent us from examining how the loss of the cholinergic cells would affect either natural or fictive swimming.

2) There seems to be an assumption that a perfect internal representation of the body wave when swimming is essential, and I'm not sure why that needs to be the case. Please explain/expand.

All movement is accompanied by an internal representation of the body, and yet it is the one sense that we know the least about (as reviewed by Tuthill and Azim). Indeed, we know so little about proprioception that it is not entirely clearly *why* or *what* is required… until it disappears. This is especially true in fish, which lack many of the proprioceptors that arose in later vertebrates, and thus we have little intuition about the possible roles or substrates of proprioception.

A major motivation of the current paper is therefore to propose a testable neuromechanical model and spur further development in this field. We build on examples from lateral line ablation and from fishes’ coordination of head and trunk motions that point to the necessity of being aware not just of the body wave but *of the body wave with respect to fluid motion*. Research in soft robotics is in fact our best indicator so far of the necessity to instantaneously judge the body configuration. In both robots and humans, visual feedback contributes to configuration estimation in the absence of proprioception. However, fluid motion is invisible, visual processing is slow, and the animal would need to be able to see its entire body (challenging in a slender fish). Feedback must therefore come from fast sensory modalities, like mechanosensation through the lateral line. We have revised the text to discuss these aspects of motor control so that the manuscript is better positioned in the literature.

3) From what I understood, in the experimental portion there were no measurements of CD, but simply the assumption that CD activity correlated with when swimming was occurring or not occurring as observed by the ventral root recordings. If that's the case, please make that clear and expand on that topic so as to justify to the non-expert reader why you made that assumption.

We regret the oversight in fully explaining this assumption. The activation of the CD during motor bouts has been extensively validated (e.g., Lunsford et al 2019, Pichler and Lagnado 2020, Roberts 1972). We have added references and text that will guide the reader to previous determinations of this known relationship.

4) I did not understand Figure 3 at all. I am not a modeler but I'd like to be able to read the results and the figure and vaguely understand what is being done and what conclusion I should draw.

We have revised this figure to build up our approach and focus on the important details. The revised figure should concisely compare the relative differences between adaptation and CD ON.

5) It was not clear to me which figures or figure panels represented computationally-generated data and which represented experimentally-generated data. Please make this abundantly clear throughout.

We have endeavoured to outline the logical flow of the manuscript culminating in our computational analysis. Nonetheless, computational statistics are a major facet of our approaches. We hope that the different approaches are now more clear. We have added an outline of our approach in the results, which should highlight the experimental and computational sections.

6) The figures are not simple. Some panels are busy and convoluted. There are often color codes, number codes, groups, symbols, shaded sections, and not all of them are clearly labeled or described in the legend. They feel too busy and demand too much of the reader. I did not feel that each panel presented one idea at a time and built logically panel by panel, but instead felt that each panel presented several overlapping ideas at a time, which made comprehension difficult. Also please revisit your color choices in Figure 2 to make them friendly to the red-green colorblind. I will list some feedback below.

We have completely revised our figures to focus on one key message in each.

We thank the reviewer for noticing this shortcoming. We have endeavoured to reselect the colour palette to accommodate all readers. We checked our figures at the website <https://www.color-blindness.com/coblis-color-blindness-simulator/> to ensure that in all figures in which colours are used, the intent is conveyed to all readers.

7) The writing style is at times excessively convoluted particularly for the non-expert. Please clarify throughout when possible.

We have completely revised and rewritten the whole manuscript. Our goal was to create a logical narrative that is visually developed through a block diagram in our new Figure 1. We expect that this will increase the clarity of our motivations and help reach a wider audience.

In general, this is a really excellent question to be asking but I cannot judge how well the methods are suited to answer it because I could not follow the presentation, and I really wanted to, because this is interesting. Making the figures and results less opaque will greatly benefit the authors as it would allow the true impact of the findings to go beyond an audience both expert in the larval zebrafish lateral line AND in computational approaches, and to reach an audience interested in sensory-motor integration and proprioception generally.

We thank the reviewer for their encouragement, and hope they find the revised version greatly improved.

Detailed points on the writing:

Line 115- rostro-caudal?

Section removed.

Line 129- Is 38 the correct reference here?

Yes, Moglie et al. studied the role of efferent neuron activity on hair cells.

Line 168- Do you mean Figure 2?

Thank you, this was an error.

Line 171-173- Is the verb missing or something I didn't get in this sentence?

The verb ‘was’ was missing from this sentence.

Line 468-469- Is there a word missing?

The word ‘system’ was missing.

All sections referred to in the preceding comments have been thoroughly rewritten and no longer apply.

Detailed points on the Figures:

Figure 1-

Ai- Do we need these? What are we gaining? If this is just to show how you measure the angle you can do that with a cartoon fish or stick fish and the angle like you show in the shaded box.

We agree and removed the unnecessary elements to streamline the figure.

Aiii- What are the dots on the side? I'm assuming the black dots on the fish below represent position of neuromasts but not explicitly stated in legend.

The dots represented the timing of the peak measured at each neuromast. This has been clarified in the revision by drawing a line to the relevant peak and focusing only on the first and last positions.

B- This was generally confusing to me and took a while to get, even though I think the concept is very good. The line placement of "Corollary discharge OFF" for example had me thinking the top line was with CD and second line was with CD OFF- the labeling was just visually confusing. I assume the thick black or red vertical lines correspond to peak firing? This is not written in the legend. Theta is not defined apart from by the cartoon. Also what is "q"? B is a really critical set up figure and I think you should make it less complicated visually if you can. This is the time in text as well to explain/convince why your idealized version is actually necessary and that when CD is off this is detrimental.

We have now thoroughly revised this figure. The figure has been simplified to better convey the key points that we wish to make. Theta is not relevant other than in this figure, and so has been removed. *q* refers to the quantiles (0.1, 0.5, and 0.9 q) that we use to define the spike response interval; we have made sure this is now defined in the legend. The thick black line corresponds to the median spike phase, which we argue should be as close to the stimulus peak (π/2) as possible.

We thank the reviewer for their insight on improving this figure and hope that the implemented changes will be convincing.

Figure 2-

Ai- I would suggest moving the Afferent trace to the bottom so that it's under the VR and the stimulus? Just an idea. Would make it easier to understand I think and to compare to Aiii.

This is an excellent suggestion and have reorganized the figure accordingly (now Figure 3).

Why is the stimulus trace black and shaded with blue instead of just blue?

We use the blue to highlight just the periods where the stimulus is on, rather than the entire sequence. We believe the revised colour scheme will be more clear.

Why is the text for Ventral root in red if the trace is black?

We have revised the figure so that only shaded intervals are coloured.

If swim and CD ON are the same, and inactive and CD OFF are the same, as indicated in the legend, why not put Swim (CD ON) on the red sections rather than put a separate label on different parts of the plot?

We have implemented this suggestion in the revised and simplified figure.

What is the necessity of having a post-swim shading? This is not clear.

We highlighted the post-swim interval so that the reader will have a reference for the post-swim interval in the former Figure 3 (now Figure 5). However, it was not necessary at this point in the text and so for clarity, we have removed it. The post-swim interval is still highlighted in Supplementary Figure 3.

Take more space, I can hardly see the scale bars, etc. It's all rather small and scrunched compared to B.

All figures have been revised with a view to clarity and reducing scrunchiness.

Aii- In the supp fig it says red is swimming but this is not stated here.

This panel has been moved to the supplementary materials and the red lines have been identified as swim bouts.

Aiii - This is complex and the legend is not sufficient. What is the main bold line and how is it calculated? I think understanding Delta E and Delta S is important and it is not intuitive as is. I suggest you break this down clearly here so that 1) B will make sense and 2) the reader can internalize it because they understand it clearly. They need to learn this new metric and have it at their fingertips going forward so it needs to be obvious here.

B- Make color choices color-blind friendly. Not everyone can distinguish these 4 colors.

Thank you. As we described above, we have revised the colour palette to accommodate all readers.

In Bi what are the large circles around each cluster? Are these calculated or approximated? No reference in the legend.

These were the confidence ellipses of the cluster centroids. The centroid is not germane to our arguments and therefore the ellipses have been removed.

This panel has been completely revised and is part of a new figure (Figure 6) that focuses solely on sensor heterogeneity. We build up to the new Figure 6 after all relevant material has been clearly presented.

By B ii and B iii I was lost.

Figure 3, 4, 5- I was completely lost by here.

We regret that the writing and figures were not of sufficient quality to permit the reviewer to follow the whole manuscript. However, we thank the reviewer for their great insight to this point. We have completely revised the text and we hope that the logical progression is now plain and will be accessible to all readers.
